# What physicians reason about during admission case review

**DOI:** 10.1007/s10459-016-9701-x

**Published:** 2016-07-28

**Authors:** Salina Juma, Mark Goldszmidt

**Affiliations:** 1 Division of Internal Medicine, Department of Medicine, Queen’s University, Kingston, ON Canada; 2 Division of Internal Medicine, Department of Medicine, University of Western Ontario, London, ON Canada; 3 Centre for Education Research & Innovation, Schulich School of Medicine & Dentistry, University of Western Ontario, Room 115, Health Sciences Addition, London, ON N6A 5C1 Canada

**Keywords:** Case review, Clinical reasoning, Clinical supervision, Clinical training, Reasoning tasks

## Abstract

Research suggests that physicians perform multiple reasoning tasks beyond diagnosis during patient review. However, these remain largely theoretical. The purpose of this study was to explore reasoning tasks in clinical practice during patient admission review. The authors used a constant comparative approach—an iterative and inductive process of coding and recoding—to analyze transcripts from 38 audio-recorded case reviews between junior trainees and their senior residents or attendings. Using a previous list of reasoning tasks, analysis focused on what tasks were performed, when they occurred, and how they related to the other tasks. All 24 tasks were observed in at least one review with a mean of 17.9 (Min = 15, Max = 22) distinct tasks per review. Two new tasks—assess illness severity and patient decision-making capacity—were identified, thus 26 tasks were examined. Three overarching tasks were identified—assess priorities, determine and refine the most likely diagnosis and establish and refine management plans—that occurred throughout all stages of the case review starting from patient identification and continuing through to assessment and plan. A fourth possible overarching task—reflection—was also identified but only observed in four instances across three cases. The other 22 tasks appeared to be context dependent serving to support, expand, and refine one or more overarching tasks. Tasks were non-sequential and the same supporting task could serve more than one overarching task. The authors conclude that these findings provide insight into the ‘what’ and ‘when’ of physician reasoning during case review that can be used to support professional development, clinical training and patient care. In particular, they draw attention to the iterative way in which each task is addressed during a case review and how this finding may challenge conventional ways of teaching and assessing clinical communication and reasoning. They also suggest that further research is needed to explore how physicians decide why a supporting task is required in a particular context.

## Background

While teaching clinical reasoning is widely acknowledged as one of the most important roles of clinical supervisors in internal medicine (Eva 2005), what physicians reason about—the reasoning tasks (e.g., identifying active issues and considering the impact of comorbid illness on management)—has received little attention (Goldszmidt et al. 2013). While multiple models exist to help teach and learn clinical tasks (e.g., taking a history, performing a physical exam) (Keifenheim et al. 2015; Kurtz et al. 2003; Lipkin et al. 1995; Makoul 2001; Silverman et al. 2005), very few models are integrated with the reasoning tasks that accompany them; the main exception are those that focus on merging clinical tasks with diagnosis such as the hypothesis-driven physical exam or history taking for the purpose of achieving a diagnosis (Hasnain et al. 2001; Yudkowsky et al. 2009). As students begin their training in clinical settings, case review becomes an important venue for learning clinical reasoning (Lingard and Haber 1999a; Spafford et al. 2006). Recent research suggests that physicians may perform multiple reasoning tasks beyond making a diagnosis while seeing a patient or reviewing a case (Goldszmidt et al. 2013). Understanding what the reasoning tasks are and how and when supervising physicians use them during case reviews has taken on greater significance in recent years as attention is drawn to the increasing complexity of hospitalized patients and to patient safety (Forster et al. 2003; Hayward et al. 2005; Nardi et al. 2007; Safford et al. 2007).

According to Goldszmidt et al. (2013), physicians may perform as many as 24 different reasoning tasks when seeing a patient. To date, medical school and residency programs have largely ignored the teaching of reasoning tasks and their relationship to the clinical tasks that accompany them (Goldszmidt et al. 2013). During the pre-clinical years, the focus is mostly on how to perform clinical tasks, such as history taking. Moreover, these are usually taught using one of the many communication models (Makoul 2001). While some communication models explicitly acknowledge the importance of teaching communication processes along with clinical content (Kurtz et al. 2003), the underlying purpose—reasoning tasks—of each clinical task, in the context of a clinical encounter, is not described. In order to address this gap, some schools have added courses on clinical reasoning (Schmidt and Mamede 2015). For the most part, the focus of these clinical reasoning courses (Schmidt and Mamede 2015) and other teaching approaches (Hasnain et al. 2001; Yudkowsky et al. 2009), not dissimilar to the clinical reasoning research itself (Bordage 1999; Bowen 2006; Higgs and Jones 2000; Montgomery 2006; Norman 2005), is on diagnostic reasoning; other reasoning tasks are largely ignored.

Patient multi-morbidity increasingly challenges physicians in their provision of patient care (Fried 2003; Marengoni et al. 2009; Safford et al. 2007). Moreover, medical teams do not always adequately address patients’ comorbid medical problems when admitting them to the hospital and consequently these patients are often readmitted soon after (Hayward et al. 2005; Pitt 2010; Vogeli et al. 2007). While several factors may contribute to medical errors and hospital readmissions, the failure to consider a patient’s chronic active diseases—a reasoning task—may play a central role (Hayward et al. 2005).

The literature suggests that trainees frequently learn reasoning tasks tacitly through case review (Lingard and Haber 1999a; Spafford et al. 2006). Case review therefore represents both threat and opportunity. For example, if during case review supervising physicians consistently fail to address things like social context or multi-morbidity, they may be inadvertently teaching trainees that these do not matter (Lingard and Haber 1999b). By contrast, addressing multi-morbidity and other relevant reasoning tasks during case review can be an effective instructional method. Having a language for explicitly drawing attention to particular reasoning tasks—why and when they are important—may also facilitate this learning (Haber and Lingard 2001; Lingard and Haber 1999a, b).

While the existing list of clinical reasoning tasks (Goldszmidt et al. 2013) offers a potential language for clinical teaching or assessment, they remain a largely theoretical entity. The only study exploring their use in clinical practice involved a think-aloud protocol of three diagnostic, video-based scenarios (McBee et al. 2015). And, while several new insights were gained about the tasks themselves and their relationships to each other, the study findings may not have been fully reflective of the use of reasoning tasks in real world settings. The purpose of the present study, therefore, was to further validate and elaborate on the original set of reasoning tasks, to explore relationships among the tasks and to identify potential new tasks as they are used in authentic real world settings. Internal medicine admission case reviews were chosen as the focus for this study because of the importance placed on clinical reasoning in internal medicine, the complexity of the patients seen, and the depth and time spent on case reviews.

## Methods

As part of a larger study focused on clinical case review and patient follow-up on an internal medicine inpatient unit (Goldszmidt et al. 2012, 2014), we performed a secondary analysis of the case review data to explore reasoning tasks as they occur during real world admission case review discussions.

### Data set

We used an existing data set that consisted of 38 admission case review discussions for patients admitted to the internal medicine inpatient teaching unit over two 8-week periods during the winter and summer 2010 (Goldszmidt et al. 2012, 2014). Data sampling was purposeful to include factors that may affect case reviews, such as weekday versus weekend admissions, attending physicians and teams to which the patients were admitted, and levels and specialty of training from junior trainees admitting patients (third-year medical students vs. first-year internal medicine vs. family medicine residents). The cases represent a variety of clinical presentations and levels of complexity that are typical of the case mix seen on the internal medicine teaching unit (Appendix 1: Table 3).

For each patient (19 in total), we collected the audio recordings for the on-call and morning case review discussions. The two sets of discussions were transcribed, creating 38 case review transcripts. The case review process took place in the usual manner. First a junior trainee completed the on-call admission assessment of the patient. The junior then presented the case to the on-call senior medical resident for review. The following morning, the junior trainee then presented again to the team, with the attending physician leading the case review. Note: In this setting, case review includes a case presentation by a junior trainee and the interruptions for teaching and guiding care that surrounds the presentation (Goldszmidt et al. 2012). For each patient, we also obtained the entire clinical record, including the junior and senior medicine resident admission notes, patient orders, progress notes, and discharge summaries. All transcripts and patient records were de-identified.

Participants included 10 attending physicians, 13 senior residents, 19 junior residents, and 14 medical students. The specific purpose of our study was not disclosed to the participants to avoid observer effect. All participants gave their consent to participate.

### Data analysis

We used a qualitative, constant comparative analysis (Charmaz 2006) to code the 38 case review transcripts for evidence of the original 24 reasoning tasks (Goldszmidt et al. 2013) (Table 1). The analysis focused on three areas: (1) Identifying reasoning tasks used, including new tasks that had not been previously identified or ones that had been identified but where the descriptions do not adequately reflect how they are used in practice; (2) exploring the relationship among tasks, (3) exploring how attending physicians brought refinements to the case review (e.g., add new reasoning tasks or elaborated on addressed reasoning tasks).

**Table 1:** Original and modified list of reasoning tasks

Original tasks	Revised tasks
Framing the encounter	
1. Identify active issues	**1. Identify active issues (overarching task)**
2. Assess priorities (based on issues identified, urgency, stability, patient preference, referral question, etc.)	2. Assess priorities (based on issues identified, urgency, stability, patient preference, referral question, etc.)
3. Reprioritize based on assessment (patient perspective, unexpected findings, etc.)	3. Reprioritize based on assessment (patient perspective, unexpected findings, etc.).
Diagnosis	
4. Consider alternative diagnoses and underlying cause(s)	4. *Consider and prioritize differential diagnoses including most likely diagnosis and most serious diagnoses to rule out*
5. Identify precipitants or triggers to the current problem(s)	5. Identifying precipitants or triggers to the current problem (s)
6. Select diagnostic investigations	6. Select diagnostic investigations *taking into account goals of care*
7. Determine most likely diagnosis with underlying cause(s)	**7. Determine most likely diagnosis with underlying cause (s) (overarching task)**
8. Identify modifiable risk factors	8. Identify modifiable *and non*-*modifiable* risk factors
9. Identify complications associated with the diagnosis, diagnostic investigations, or treatment	9. Identify complications associated with the diagnosis, diagnostic investigations, or treatment
10. Assess rate of progression and estimate prognosis	10. Assess rate of progression, *response to treatment* and estimate prognosis *and length of stay*
11. Explore physical and psychosocial consequences of the current medical conditions or treatment	11. Explore physical and psychosocial consequences of the current medical conditions or treatment
Management	
12. Establish goals of care (treating symptoms, improving function, altering prognosis or cure; taking into account patient preferences, perspectives, and understanding)	12. Establish goals of care (treating symptoms, improving function, altering prognosis or cure, taking into account patient preferences, perspectives, and understanding)
13. Explore the interplay between psychosocial context and management	13. Explore the interplay between psychosocial context and management
14. Consider the impact of comorbid illnesses on management	14. Consider the impact of comorbid illness on management
15. Consider the consequences of management on comorbid illnesses	15. Consider the consequences of management on comorbid illnesses
16. Weigh alternative treatment options (taking into account patient preferences)	16. Weigh alternative treatment options (taking into account patient preferences, *expert opinion, evidence based practice, risks/benefits and attending preferences*)
17. Consider the implications of available resources (office, hospital, community, and inter- and intraprofessionals) on diagnostic or management choices	17. Consider the implications of available resources (office, hospital, community, and inter- and intraprofessionals) on diagnostic or management choices *taking into account most appropriate service to admit patient to and requirements for discharge planning*
18. Establish management plans (taking into account goals of care, clinical guidelines/evidence, symptoms, underlying cause, complications, and community spread)	**18. Establish management plans (taking into account goals of care, clinical guidelines/evidence, symptoms, underlying cause, complications, and community spread) (overarching task)**
19. Select education and counseling approach for patient and family (taking into account patients’ and their families’ levels of understanding)	19. Select education and counselling approach for patient and family (taking into account patients’ and their families’ level of understanding)
20. Explore collaborative roles for patient and family	20. Explore collaborative roles for patient and family

Data analysis was performed by the two investigators, an attending physician in General Internal Medicine with a PhD in health professions education (MG) and a third-year resident in internal medicine (SJ), thus allowing the inclusion of perspectives from both an attending physician and a senior resident. The primary coding was done by SJ, who met with MG on a regular basis to review the coding, discuss task definitions, and reconcile any discrepancies in what constituted a legitimate instance of a reasoning task. Prior to any coding, SJ read through the entire transcript and associated clinical notes. A section of the transcript was coded as representing a particular reasoning task when participants made explicit reference to the task or to reasoning about a particular piece of data or if the reasoning task could be inferred in the context of the case and subsequent dialogue. Inferred tasks were typically not coded until at least a second or third reading of the transcript as well as review of the corresponding hospital records that followed. Annotations and memos were made throughout to mark interesting or unclear examples.

Individual portions of text could contain more than one reasoning task. Moreover, there were often multiple utterances of the same reasoning task repeated within several portions of the text, without representing a distinct task, so the number of codes per task did not represent the true frequency of each task. In Table 2, each distinct task was only counted once per case even if it may have occurred in numerous places in the text. Each coded reasoning task was also coded based on which participant was expressing the task (attending physician, senior resident or junior trainee).

**Table 2:** Reasoning tasks in relationship to the overarching tasks, number of cases they occurred in and number of cases they were refined by the attending in

Reasoning tasks	Overarching tasks				Number of cases task occurred in (max = 19)	Attending refined^b^
	Identify active issues	Determine the most likely diagnosis and underlying cause	Establish management plans	Reflection^a^		
1. Overarching task: Identify active issues	X				19 (100 %)	13 (68.4 %)
2. Assess priorities (based on issues identified, urgency, stability, patient preference, referral question, etc.)	X	X	X		19 (100 %)	13 (68.4 %)
3. Reprioritize based on assessment^a^	X	X	X		19 (100 %)	13 (68.4 %)
4. Consider and prioritize differential diagnoses including most likely diagnosis and most serious diagnoses to rule out^a^		X			19 (100 %)	6 (31.6 %)
5. Identifying precipitants or triggers to the current problem (s)	X	X	X		19 (100 %)	2 (10.5 %)
6. Select diagnostic investigations taking into account goals of care^a^		X			18 (94.7 %)	3 (15.8 %)
7. Overarching task: Determine most likely diagnosis with underlying cause(s)		X			19 (100 %)	12 (63.2 %)
8. Identify modifiable and non-modifiable risk factors	X	X	X		10 (52.6 %)	3 (15.8 %)
9. Identify complications associated with the diagnosis, diagnostic investigations, or treatment		X			19 (100 %)	3 (15.8 %)
10. Assess rate of progression, response to treatment and estimate prognosis and length of stay	X	X	X		19 (100 %)	7 (36.8 %)
11. Explore physical and psychosocial consequences of the current medical conditions or treatment	X	X	X		12 (63.2 %)	1 (5.3 %)
12. Establish goals of care (treating symptoms, improving function, altering prognosis or cure, taking into account patient preferences, perspectives, and understanding)	X	X	X		9 (47.4 %)	2 (10.5 %)
13. Explore the interplay between psychosocial context and management	X	X	X		18 (94.7 %)	3 (15.8 %)
14. Consider the impact of comorbid illness on management	X	X	X		15 (78.9 %)	6 (31.6 %)
15. Consider the consequences of management on comorbid illnesses	X	X	X		10 (52.6 %)	6 (31.6 %)
16. Weigh alternative treatment options (taking into account patient preferences, expert opinion, evidence based practice, risks/benefits and attending preferences)			X		17 (89.5 %)	6 (31.6 %)
17. Consider the implications of available resources (office, hospital, community, and inter- and intraprofessionals) on diagnostic or management choices taking into account most appropriate service to admit patient to and requirements for discharge planning	X		X		10 (52.6 %)	4 (21.1 %)
18. Overarching task: Establish management plans (taking into account goals of care, clinical guidelines/evidence, symptoms, underlying cause, complications, and community spread)			X		19 (100 %)	14 (73.7 %)
19. Select education and counselling approach for patient and family (taking into account patients’ and their families’ level of understanding)			X		4 (21.1 %)	2 (10.5 %)
20. Explore collaborative roles for patient and family			X		2 (10.5 %)	1 (5.3 %)
21. Determine follow up, monitoring and consultation strategies (taking into account urgency, how pending investigations/results will be handled)^a^			X		17 (89.5 %)	8 (42.1 %)
22. Determine what to document and who should receive documentation			X		4 (21.1 %)	3 (15.8 %)
23. Assess severity	X	X	X		16 (84.2 %)	4 (21.1 %)
24. Assess decision-making capacity			X		4 (21.1 %)	1 (5.3 %)
25. Identify knowledge gaps and establish personal learning plans				X	3 (15.8 %)	2 (10.5 %)
26. Consider cognitive and personal biases that may influence reasoning				X	1 (5.3 %)	1 (5.3 %)

The X in each box indicate that the task could be used to support that overarching task

^a^While the data for reflection only tentatively support its inclusion as an overarching task, it is included in the table to support the representation of tasks 25 and 26

^b^Represents the number of cases where the attending refined each reasoning task or added the task as a new one for the team to consider

The 38 transcripts were coded and recoded by SJ in consultation with MG using an iterative process. All transcripts were reviewed at least five times. Evidence of reasoning that did not fit the original task definitions were initially coded as “Other.” These were then analyzed separately and as a group until consensus was developed between the two investigators regarding their fit into an existing task definition. Task definitions were then modified and elaborated to account for these. The remaining “other” coded tasks were then analyzed to determine what type of reasoning task they represented and new definitions were developed for these. The data set was then coded again to confirm and verify coding as well as to look for evidence of the newly defined reasoning tasks. Coding was done using NVivo version 10, qualitative data analysis software.

Several strategies were used to explore the relationship between tasks. For each transcript, a high-level summary was created that captured the salient features of the case review and the reasoning tasks addressed (Charmaz 2006). Similarly, a summary was drawn for each reasoning task to describe where the reasoning tasks occured during the case review and which other tasks tended to overlap with that task. Using NVivo’s different display properties, we also reviewed the extent to which different codes overlapped with each other within and across cases and at different sections within a case (e.g., past medical history, physical exam etc.). This process generated a group of clusters around which multiple codes typically overlapped. Through our cross case analysis, where patterns of clusters were explored between different cases, we were able to identify our final framework of overarching and supportive tasks.

We also used NVivo’s display properties to review and compare the reasoning tasks expressed by the senior residents and attending physicians during the same case in order to explore what the attending physicians added to the case review that the senior resident had not already addressed. Tasks were considered refined by the attending if there was no evidence to suggest the senior resident had already addressed the reasoning task or if the attending contribution substantively altered how the task was being carried out.

## Results

The case reviews included a range of clinical presentations and active issues that elicited the use of diverse sets of reasoning tasks, as illustrated in Appendix 1: Table 3. Junior trainees and their supervisors engaged in a mean of 17.9 (SD = 1.8; Min = 15, Max = 22) distinct reasoning tasks per case review. All 24 original tasks were observed during at least one case review (Table 1). Additionally, two new reasoning tasks were identified (Table 1): Assess severity, when determining the magnitude of the disease (Task 23; present in 16 cases), and assess decision-making capacity, when determining the patient’s ability to make decisions (Task 24; evident in 2 cases) (Table 1). We were also able to refine the descriptions for 7 of the original 24 reasoning tasks based on what we observed in these real world inpatient case reviews (Table 1).

**Fig. 1:**
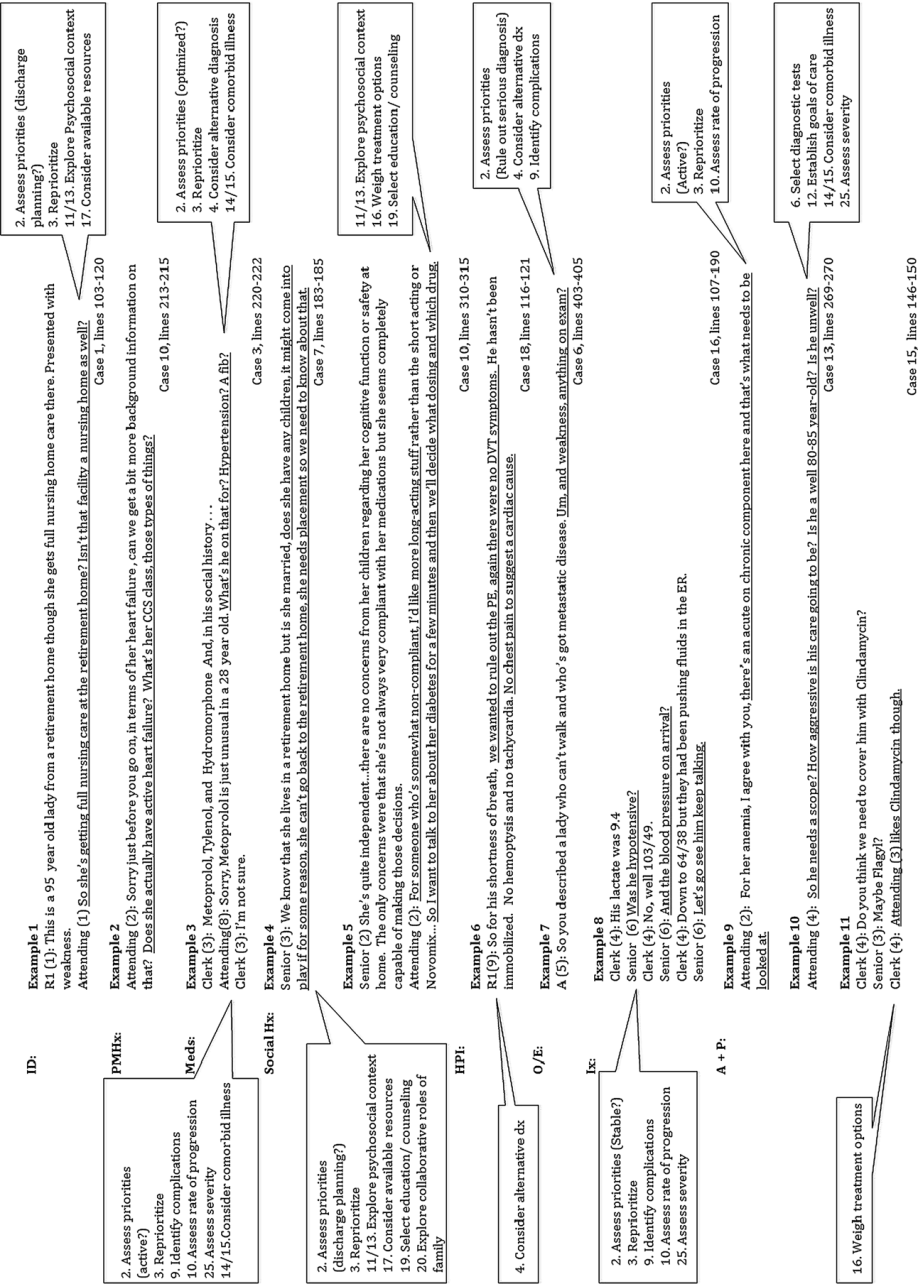
Case review examples in relation to reasoning tasks

Three of the 26 clinical reasoning tasks represented overarching tasks under which the other tasks were subsumed, that is: “identify active issues,” “determine the most likely diagnosis and underlying cause,” and “establish management plans.” These three reasoning tasks were addressed in all cases and were strongly inter-related; once an issue was identified as active, it would, at a minimum, require that the team establish a management plan for it. A fourth possible overarching task—reflection—was also identified but only observed in four instances across three cases. In all cases, it appeared to reflect a form of self-monitoring where a knowledge gap was identified and a plan for addressing it was explicitly stated. Because it was visible in so few cases, it is not possible to determine if it represents an overarching task that our methodology simply failed to fully capture or if it represents a set of tasks that individuals only occasionally attend to. The remaining reasoning tasks served to support and refine the three overarching tasks. Moreover, these supporting tasks were not unique to any one overarching task. For example, in case 14, we found that task 8, “identify modifiable risk factors”, could be used to support the diagnosis (poorly controlled diabetes as a risk factor for an infected ulcer), to identify active issues (diabetes as an additional active issue), and to establish a management plan (altering insulin regimen, selecting counseling). The supporting tasks also appeared to be context specific, occurring only when relevant to the specifics of the case. For example, the reasoning task “establish goals of care” became relevant in elderly patients with multiple comorbidities (Fig. 1-Example 10). This meant that some tasks occurred relatively infrequently whereas others occurred in all or nearly all cases, as shown in Table 2.

**Fig. 2:**
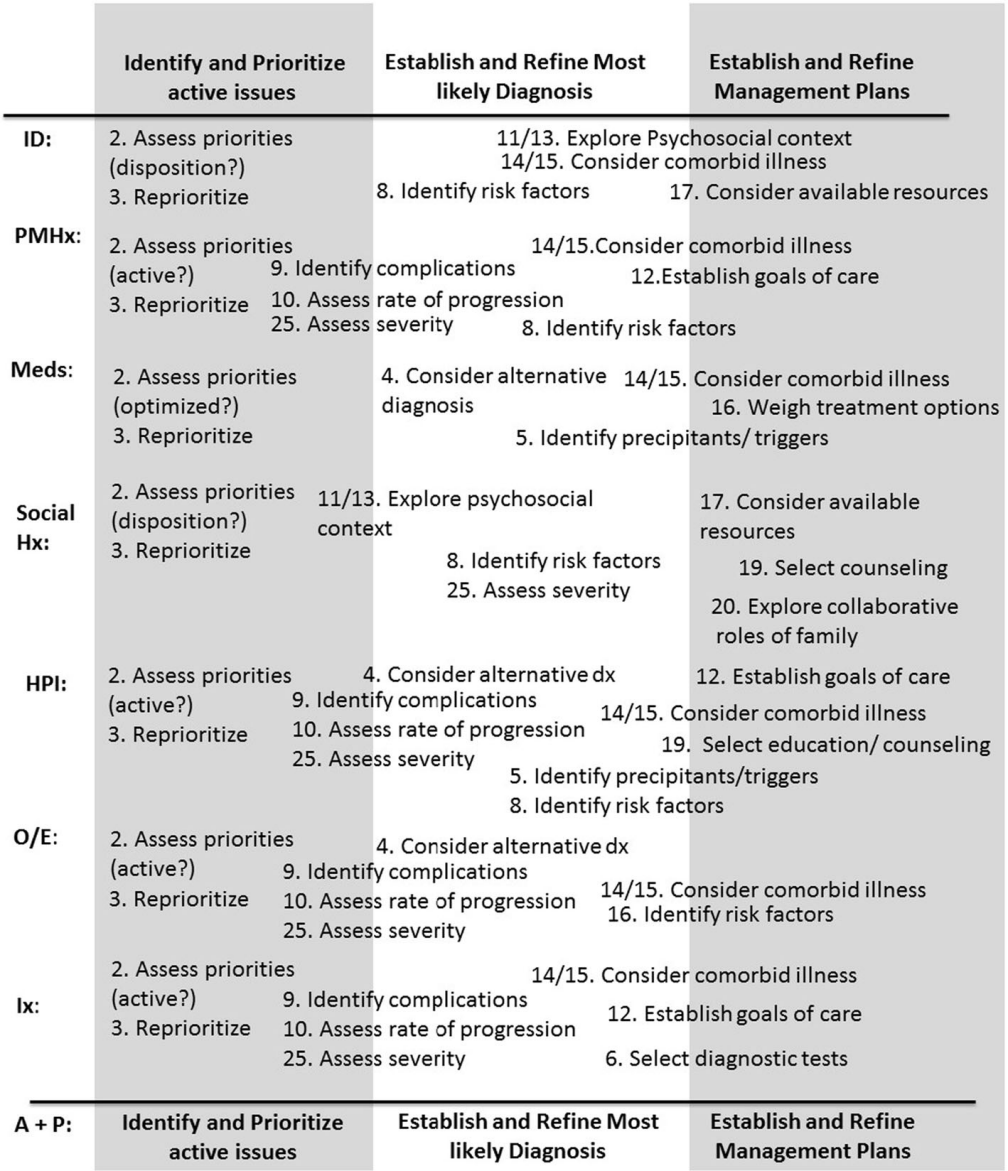
Schematic representation of the three oveararching tasks, the reasoning tasks in relation to the overaraching tasks and timing during the encounter when they could take place

Rather than occurring at discrete moments, all three overarching tasks appeared to be addressed throughout the case review process in an iterative manner, starting as early as the patient identification and continuing on through to the end of the assessment and plan (Fig. 2). For example, while it was not explicit, during the review of patient identification, the attending physicians often explored a patient’s social situation in order to determine whether discharge planning would be an active issue (Fig. 1-Example 2). This could be re-evaluated and built upon throughout the remainder of the case review. For example (Fig. 1-Example 4), in the review of the social history, the senior resident explored the social situation with respect to the family supports (Task 20) and the available resources at the retirement home (Task 17), and determined that the patient’s discharge location may be an issue.

In the next section, we will present the three overarching tasks and describe the supporting tasks involved and any modifications to the original task definitions we observed. We will also explore how the attending physicians refined the reasoning tasks or added new reasoning tasks to the case review (Table 1).

### Identifying active issues

There was evidence of “identifying active issues” at all stages of the case review process, starting from the review of the patient identification through to the discussion of the assessment and plan (Fig. 2). In the original classification (Goldszmidt et al. 2013), three tasks (1. Identify active issues, 2. Assess priorities, 3. Reprioritize) had been placed under the category title “Framing the Encounter.” These three tasks did not, however, appear to frame the encounter as observed during case reviews. Rather, the standard presentation format—identification, past medical history etc.—served this framing function. The original three tasks along with several other tasks (Tasks 5, 8, 10–15, 17, 25) all served to help the team to identify and prioritize the active issues list (Table 2). For example, in Case 11, when identifying active issues, the attending assessed the rate of progression and severity of the patient’s chronic anemia (Tasks 10 and 25) and determined that it should be addressed (Fig. 1-Example 9).

Attending physicians helped the team to refine their thinking around this task in 13 of the 19 cases, as illustrated in Table 2. Examples of areas where they did this include: probing more deeply the patient’s social setting and supports to determine whether discharge placement would be an active issue (4 cases, e.g. Fig. 1-Example 2); and refining the active issues list to consider the patients’ chronic medical issues and determining whether they needed to be actively addressed during their hospitalization (5 cases, Fig. 1-Example 1).

### Determining most likely diagnosis and underlying cause

As with the previous overarching task, there was evidence of this overarching task at all stages of the case review (Fig. 2). The original tasks under the Diagnosis category (Tasks 4–11) along with several other tasks (Tasks 2, 3, 12–15, 25) served to support and refine the most likely diagnosis (Table 2). For example, in case 10, when admitting a patient with a diabetic foot ulcer, the team considered: the patient’s poorly controlled diabetes as a modifiable risk factor and a comorbid illness to be addressed (Tasks 8, 14, 15); the history of trauma as a precipitant (Task 5); and the possibility of osteomyelitis as a complication (Task 9). They also prioritized osteomyelitis as a complication that needed to be ruled out (Task 2).

Based on the current findings, four of the supporting tasks required modification and elaboration (Table 1). Two salient examples are the modifications made to Tasks 4 and 10. Task 4, originally related to considering the differential diagnosis, was expanded and modified to reflect the need to both consider and prioritize based on the most likely diagnosis and the most serious diagnoses to rule out. This is exemplified in case 7 where the senior resident discussed the differential for ankle swelling and explained that gout was the most likely diagnosis but septic arthritis was the most important to rule out. Task 10, originally focused on rate of progression and prognosis, was modified to include response to treatment. For example, in Case 19, the team starts IV fluids for acute kidney injury (AKI) and uses the rate of improvement to confirm that the AKI was secondary to dehydration.

While attending physicians discussed this overarching task at length with their team in all cases, this led to the refinement of their thinking for 12 cases (Table 2); for the most part, the on-call team had typically considered most of the relevant issues related to this overarching task. Examples of areas where refinements did occur included assessing the rate of progression of disease (7 cases) and exploring the possibility of serious alternate diagnoses (6 cases). Figure 1, example 8 is a good example of the attending helping the team to refine their thinking with regards to ruling out serious alternative diagnoses.

### Establishing management plans

While the management plan was primarily discussed in the assessment and plan portion of the case review, there was evidence of this overarching task occurring at all stages of the case review (Fig. 2). The original tasks under the Management category (Tasks 12–20) along with several other tasks (Tasks 2, 3, 5, 8, 10, 11) served to support and refine the management plan (Table 2). For example, in Case 16, the senior resident assessed severity (Task 25) of the patient’s sepsis after hearing that the patient’s blood pressure was low and triaged the case (Tasks 2 and 3) and the management as more urgent (Fig. 1-Example 7).

Based on the study findings, three of the supporting tasks required modification and elaboration (Table 1). Tasks 17 and 16 are both good examples of these. When considering available resources (Task 17), teams also appeared to take into consideration the patient’s existing supports and required level of care in order to determine where the patient would be discharged to following hospitalization (Fig. 1-Examples 1 and 4). Team members considered several factors when weighing treatment options (Task 16), including expert opinion, evidence-based practice, risks and benefits, and attending preferences (Fig. 1-Example 11).

Attending physicians refined their thinking about management plans in 14 cases. Examples of refinements included exploring the patient’s level of care to determine possibilities for discharge location (4 cases, Fig. 1-Example 2); addressing goals of care when discussing investigation and management plans (2 cases, Fig. 1-Example 10); considering the impact of comorbid illness on management (6 cases), weighing alternative treatment options (6 cases), taking into account psychosocial contexts (3 cases, Fig. 1-Example 6), and determining the need for follow-up or consultation (8 cases).

## Discussion

The purpose of this study was to validate the initial set of 24 reasoning tasks (Goldszmidt et al. 2013) and to explore how and when they are used during admission case reviews. We were able to validate and elaborate on the original set of tasks, explore relationships between tasks, and describe how attending physicians refined and contributed to the reasoning tasks addressed by the on-call team during case reviews. These results have implications for education, clinical practice, and research.

### Validation and elaboration of reasoning tasks

Across the data set, we were able to identify multiple instances of reasoning around all 24 of the original reasoning tasks. This adds to McBee et al.’s (2015) identification of 14 of the 24 tasks during a diagnostic think aloud protocol. In that study, they also identified several possible modifications to the original task descriptions. As seen in Table 1, most of their findings were confirmed in our study resulting in several modifications to task descriptions. We also identified two new tasks that could not be incorporated into the original task definitions. This allowed us to expand and elaborate on our original list to create a more comprehensive set of reasoning tasks that better reflect real world practices, at least during case reviews (Table 1).

### Relationships among tasks

We identified a high degree of overlap and interaction among the reasoning tasks, with three overarching tasks being dominant and a possible fourth acting as a self-monitoring task (reflection). The overarching tasks provided a structure for the supporting tasks. This is in contrast to the original classification that placed the tasks in one of four categories. Our current findings support a greater degree of fluidity among tasks. Depending on the context, a given supporting task can serve more than one overarching task. Moreover, depending on the problem(s) in a particular case, nuances were explored by drawing on a variety of supporting tasks; as such, the supporting tasks were very context specific, occurring only when relevant to the specifics of the case.

As proposed in the original study, the sequence of reasoning tasks was found to be non-linear. While this non-linearity was also identified by McBee et al. in their study (McBee et al. 2015), at times, the tasks appeared to be repetitive. In the present study, the tasks were also revisited at multiple points during a case review. However, rather than being viewed as repetitive, they were iterative and cumulative. For example, during the patient identification, an attending may probe tentatively at the patient’s living situation which, as the case unfolds, may or may not lead to the identification of where to discharge the patient to post-hospitalization.

### Attending’s refinement of reasoning during case reviews

Attending physicians probed and refined around all three overarching reasoning tasks. This was especially true when identifying active issues and establishing management plans. We postulated from this that attending physicians did not refine as many tasks related to diagnosis because these had generally been thoroughly addressed overnight by the senior resident; refinements here largely focused on broadening the differential diagnosis. By contrast, attending physicians supported more extensive refinements related to: identifying chronic active medical issues that need to be addressed; weighing alternative treatment options; considering the impact of patient and family preferences/goals of care on management; psychosocial context and how it may influence management choices; follow up and consultation strategies; and available resources and their impact on planning around patient disposition. Focusing on these types of refinements in future research may provide insight into expertise effects in relation to reasoning tasks thus extending our understanding of reasoning expertise beyond diagnostic reasoning.

### Relationship to theories of clinical communication

Our findings also have implications for the case review genre itself. Clinical encounters, case reviews, and their associated documentation all follow a particular sequence (i.e., patient identification, reason for assessment, past medical history, history of present illness etc.). Though the sequence may be institution specific, deviations from this sequence during case reviews, for example, are typically redirected back to the expected sequence (Goldszmidt et al. 2012). While having such a sequence is necessary from a communication and collaboration perspective (Devitt 2004), we found that the case presentation format was frequently framed based on this sequence rather than on the reasoning tasks and what was necessary to achieve them. According to Klein, “sensemaking is a process of framing and reframing, of fitting data into a frame that helps us filter and interpret the data while testing and improving the frame and cyclically moving forward to further adapt the frame. The purpose of a frame is to define the elements of the situation, describe the significance of these elements, describe their relationship to each other, filter out irrelevant messages, and highlight relevant messages.”(Klein et al. 2007, p. 119). Three parts of the presentation are particularly salient in this regard: the chief complaint (sometimes referred to as the reason for referral), the past medical history and the history of present illness. In all three of these sections, we identified meaningful “sensemaking” and “reframing” by the attending physicians during the case presentation—what we refer to as refinements above. With the increasing complexity of patients seen in the inpatient setting (Forster et al. 2003; Hayward et al. 2005; Nardi et al. 2007; Safford et al. 2007), it may well be that a modification to the case presentation and documentation genres could help trainees to recognize the need to address a broader set of reasoning tasks during their patient encounters. For example, past medical history could be separated, as Weed has suggested (Weed 1968), into active and inactive components; this would help trainees recognize that some of the patient’s chronic active issues will need to be thought through in the context of their current presentation. Similarly, the history of present illness could be re-titled to history of active problems. This might help trainees to recognize the need to explore more than just the chief complaint.

Likewise, there are implications for theories and models of clinical communication. In the original classification (Goldszmidt et al. 2013), based on the Calgary Cambridge model (Kurtz et al. 2003) and other similar training guides (Makoul 2001; Lipkin et al. 1995), it was indicated that the first step a student takes is to initiate the encounter and identify active issues. Our findings allows us to further elaborate on this model by emphasizing to trainees the iterative and cumulative process of using reasoning tasks that occur throughout all stages of the clinical encounter; in particular, that only a tentative agenda can be negotiated upfront as, in some contexts, identification of active problems occurs throughout the encounter. These findings may also apply to other clinical contexts, like outpatient and emergency room visits. Further research is needed to specify how a modified model of communication can be taught effectively, how it would impacts performance and how context shapes its use.

### Implications for education, clinical practice, and research

In the original study (Goldszmidt et al. 2013), several applications of the reasoning tasks had been suggested for teaching and education research. Findings from the present study allow us to expand on those as well as gain new insights.

The reasoning tasks we observed during case review discussions were often inferred rather than explicit. It was unclear whether junior trainees recognized the underlying reasoning and relevance of the questions asked when attending physicians and senior residents probed different aspects of the case. Prior research has also shown that the feedback given to trainees during case presentations is often implicit and without context (Lingard and Haber 1999a; Spafford et al. 2006). For example, when an attending asks the trainee to skip over the review of systems or social history during a particular case, are they doing so because it is not relevant for this case, never relevant during case review or is there simply no time for it at this moment in time? Many attending physicians have only a tacit understanding of reasoning tasks (Schön 1983). Our list of 26 reasoning tasks can provide a more comprehensive language to discuss reasoning tasks and thus make reasoning more explicit and foster a deeper understanding of clinical reasoning in their trainees during case reviews.

Our findings also have implications for trainee evaluation and assessment. According to Irby, attending physicians during case reviews should diagnose both the patient and the learner (Irby 1992). Based on our findings, we suggest that the focus on diagnosing the patient can be expanded to include the other dominant tasks (e.g., identifying active issues and refining the management plan), along with their supportive tasks. There were several reasoning tasks, often outside of diagnosis, that were added by the attending physicians that the junior and senior residents did not appear to consider fully. Expanding Irby’s model would allow us to better identify patterns and gaps of individual trainees about specific reasoning tasks in order to give them more concrete feedback. As we look toward developing specific competencies when diagnosing the learner, future research is needed to explore what would be expected with regard to reasoning tasks as trainees mature to more senior roles and whether this improves assessment.

The complexity of the reasoning tasks we observed in this study also adds insights into implications for cognitive load and medical errors in clinical practice. Durning and colleagues found that multiple different contextual factors in a particular case could increase cognitive load and negatively affect physician performance as measured by post encounter assessment forms (Durning et al. 2011, 2012). Similarly, we expect that physician performance may be hampered when engaging in encounters or case reviews that require multiple reasoning tasks to be used during a complex iterative process (e.g., a patient with multiple comorbidities and active problems). This calls for more research to explore how physicians handle the complexity of reasoning tasks and whether certain tasks are omitted due to increased cognitive load. This also applies to medical errors research that could explore which reasoning tasks are most likely to be omitted to the detriment of patient care in specific contexts.

In the original study (Goldszmidt et al. 2013), it was suggested that the list of reasoning tasks could be used as a reflective tool for clinicians in practice. Our findings further reinforce this. In addition to reflecting on the particular reasoning tasks they do and do not use well, clinicians could also reflect on the extent to which they engage in the complex process we found. While it may be tempting to use our list of reasoning tasks as a checklist to improve clinical practice or reduce medical errors, we would caution against this. In our study, many of the supporting tasks were not relevant to every case. This relates to the concept of case specificity, which has been shown to be an integral part of diagnosis and management (Norman et al. 2006). Given the complexity of the reasoning tasks and the notion of case specificity, a checklist of reasoning tasks would be too simplistic. However, a very viable future research direction could be to explore how novices, intermediates, and experts differ in the reasoning tasks they use during an encounter.

### Limitations

While our study has numerous implications for both case reviews and clinical encounters, we did not study clinical encounters broadly. We suspect that case reviews represent but a limited aspect of clinical encounters in general. Thus further research is needed to observe how physicians use reasoning tasks during different types of clinical encounters. Similarly, our study was focused on patients being admitted to an inpatient internal medicine teaching unit. These patients are typically elderly and tend to have a high degree of multi-morbidity. Future research should therefore explore how physicians use reasoning tasks in other contexts.

When exploring which reasoning tasks senior residents appeared to omit, it was difficult to be certain that these reasoning tasks were not considered at all rather than simply not verbalized. Senior residents may have omitted certain reasoning tasks due to legitimate time constraints overnight rather than a failure to recognize their importance. Therefore, these omissions do not necessarily reflect a lack of understanding or ability. Similarly, while we did observe four instances of reflection, it does not appear to be the norm that these are shared during case reviews; rather, it may be something that each individual is doing during more private moments. Finally, we did not explore the potential effects of providers’ demographic characteristics such as gender and age on clinical reasoning. This may be an interesting area for future exploration.

In conclusion, the further validation and elaboration of reasoning tasks in the real world setting of internal medicine case reviews revealed a rich and complex framework for clinical reasoning. Insights gained from this study provide an opportunity for reconsidering how we teach and study clinical communication and reasoning.

## Appendix 1

See Table 3.

**Table 3:** Brief description of the 19 cases and the total number of distinct reasoning tasks addressed per case

ID	Age range	Reason for admission	Other active issues	Number of reasoning tasks per case (Max = 26)
1	90–100	Dehydration secondary to diarrhea	End stage dementia, acute renal failure, hypernatremia, palliation	20
2	80–90	UTI and fall with knee trauma	Query syncope, atrial fibrillation, dementia, delirium, hypokalemia	22
3	20–30	Febrile neutropenia	Dermatomyositis, gastroenteritis, pancytopenia, B12 deficiency, iron deficiency, feeding tube	19
4	90–100	Acute renal failure and hypernatremia secondary to dehydration	Urinary tract infection, dementia, delirium, hypotension, bradycardia, anemia, conjunctivitis	18
5	60–70	Aspiration pneumonia	Dementia with behavioural issues	20
6	70–80	Pneumonia	Metastatic non-small cell carcinoma, gastro-intestinal bleed, malnutrition, hypokalemia, hypomagnesimia	18
7	60–70	Gout	Type II diabetes, atrial fibrillation on coumadin, sleep apnea, pulmonary HTN, COPD, hypokalemia	20
8	60–70	Pancreatitis	Alcohol withdrawal, narcotic dependency, dilated biliary duct, Type II diabetes, hypothyroidism	17
9	70–80	Drug induced rash and hyponatremia	Tricuspid regurgitation with pedal edema, atrial fibrillation on coumadin, Type II diabetes	17
10	80–90	Cellulitis of foot	Atrial fibrillation on coumadin, diarrhea, angina, peripheral edema, Type II diabetes, hyponatremia, hypothyroidism	19
11	60–70	COPD exacerbation secondary to pneumonia	Anemia, elevated creatinine kinase, Type II diabetes, nausea and depression	16
12	80–90	Decrease level of Consciousness and urinary tract infection	Pneumonia, dementia, acute renal failure, hypotension, anemia (query gatrointestinal bleed)	15
13	80–90	Acute on chronic anemia	Query pneumonia, coronary artery disease, diabetes, new heart murmur, prostate cancer	19
14	50–60	Infected diabetic foot ulcer	Type 1 diabetes, COPD, congestive heart failure, nausea and vomiting, elevate INR, Hypokalemia	18
15	50–60	Aspiration pneumonia with secondary empyema	Narcotic abuse and overdose, hypotension, COPD, acute renal failure, iron deficiency anemia, tricuspid regurgitation	15
16	80–90	Sepsis secondary to urinary tract infection and bacteremia	Bladder cancer, Acute renal failure, hydronephrosis, Congestive Heart Failure, Gastrointestinal bleeding, atrial fibrillation on coumadin	18
17	70–80	Partial small bowel obstruction	IgA multiple myeloma with gastrointestinal tract involvement, acute renal failure, anemia, B12 deficiency, angina, congestive heart failure, hypotension, hypokalemia, chronic pain	17
18	60–70	Pneumonia with parapneumonic effusion	Type II diabetes, atrial fibrillation on ASA, obstructive sleep apnea, cardiomegaly	15
19	70–80	Post-operative pulmonary embolus	New onset atrial fibrillation, acute renal failure, cellulitis, urinary tract infection, hypothyroidism	18
			Mean	17.94
			Max	22
			Min	15
